# Epilepsy

**Published:** 1853-10

**Authors:** 


*Epilepsy.*—In a recent work by M. Herpin, which received a prize from the Institute of France, the prognosis and treatment of epilepsy are ably discussed. The remedies which M. Herpin found most successful were the salts of zinc, the ammonio-sulphate of copper, valerian, and an umbelliferous plant, the Selinum palustre. The oxide of zinc was exclusively employed in forty-two cases. Thirty-one of these had suffered from less than 100 fits; twenty-six were cured, and five were incurable; five had had less than 500 fits; two were cured, and three were incurable; six had had over 500 fits, and were all incurable. The oxide of zinc was commenced in quantities of from six to eight grains daily, given in divided doses, one hour after each meal. It was augmented two grains daily until the dose reached forty-five grains. With regard to the time when the remedy may be supposed to have had a fair trial, the following rules are laid down by M. Herpin: In children under one year, seventy-five grains should be administered before the remedy is given up. In cases over two years, and in which the prognosis is only moderately favorable, (cases of more than 100 attacks,) as much as four ounces must have been given during the treatment before another remedy is employed.—*From Virginia Med. and Surg. Jour.*

